# Determining factors of diuresis in chronic kidney disease patients initiating hemodialysis

**Published:** 2015

**Authors:** A-M Nechita, D Rădulescu, I Peride, A Niculae, O Bratu, D Ferechide, A Ciocâlteu, IA Checheriță, D Mischianu

**Affiliations:** * Department of Nephrology and Dialysis, “Sf. Ioan” Clinical Emergency Hospital, Bucharest, Romania; ** Clinical Department No. 3, “Carol Davila” University of Medicine and Pharmacy Bucharest, Romania; *** Department of Urology, “Dr. Carol Davila” Central Military Emergency University Hospital, Bucharest, Romania; **** Department of Physiology I, Clinical Department No. 2, “Carol Davila” University of Medicine and Pharmacy Bucharest, Romania

**Keywords:** stage 5 chronic kidney disease, residual diuresis, hemodialysis

## Abstract

**Background:** Patients with stage 5 chronic kidney disease (CKD) begin chronic hemodialysis with variable diuresis levels correlated to a comparable low glomerular filtration rate. Residual diuresis influences long-term evolution of the hemodialyzed patient, modifying the prognosis even if optimal Kt/ V is achieved.

**Aim of the study:** The present study emphasizes the main determining factors of diuresis in a cohort of stage 5 CKD subjects at the beginning of hemodialysis.

**Material and methods:** 216 patients with stage 5 CKD starting chronic hemodialysis were included in the study, and were grouped according to their residual diuresis: group A (urine output ≤ 500 mL/ day); group B (urine output between 500–1200 mL/ day); group C (urine output ≥ 1200 mL/ day).

**Results:**Glomerular etiology, cardiac systolic dysfunction, severe malnutrition, emergency dialysis initiation and lack of permanent vascular access were proved to be associated with significant low diuresis. Age, gender, estimated glomerular filtration rate (GFR) and the presence of systemic hypertension did not influence the amount of daily diuresis.

**Conclusions:** In CKD stage 5 patients, residual diuresis presents large variations in conditions of comparable low GFR. Factors influencing residual diuresis may be distinct from those that influence residual GFR.

## Introduction

Statistic data highlighted the importance of preserving residual renal function (RRF) at the beginning of dialysis. Increasing dialysis efficiency, in terms of increasing the dose of dialysis (Kt/ V) and/ or increasing intradialysis ultrafiltration is not associated with the same favorable effects [**1**-**4**]. Preserved RRF is accompanied by multiple benefits: better removal of uremic toxins [**3**,**5**-**8**], increased phosphate urinary excretion and superior handling of secondary hyperparathyroidism [**3**,**5**-**7**,**9**], reduced cardiovascular complications and better volume control [**3**,**5**-**7**,**10**-**12**], improved nutritional markers [**3**-**7**,**13**], decreased need for erythropoiesis stimulating agents [**3**,**5**-**7**], reduced proinflammatory state [**3**,**5**-**7**,**14**,**15**], improved quality of life [**3**,**5**-**7**] and reduced all-cause mortality [**2**,**3**,**5**-**7**,**16**-**18**].

The studies that have been conducted used – as a measure for residual renal function – either the residual glomerular filtration rate (estimated with different formulas) (GFR), or the residual urine output [**19**]. Therefore, these two entities could have been considered similar. Additionally, several other researches emphasize the fact that in uremia, urine formation depends on several factors [**20**,**21**], being determined not only by GFR alone, but also by the tubular reabsorption activities and the osmotic loads in the blood [**20**-**22**]. Consequently, in advanced renal failure, accompanied by low residual GFR, patients might present different diuresis, from oligoanuria to polyuria. 

Therefore, considering these findings and our own observations, as well, we concluded that residual diuresis at the beginning of dialysis has a variable importance in the evolution of patients, apart from the residual GFR. We conducted a study, part of a larger research on the importance of diuresis in hemodialysis (HD) population, on the factors that might influence the residual diuresis at the initiation of hemodialysis.

## Material and methods

216 patients with chronic kidney disease (CKD) stage 5 beginning chronic HD were included in the study. Patients with uncertain diagnosis (acute-on-chronic renal failure and acute kidney injury) were excluded. The patients were divided in three groups, depending on their residual diuresis: group A (urine output ≤ 500 mL/ day); group B (urine output between 500–1200 mL/ day); group C (urine output ≥ 1200 mL/ day).

Several factors were noted in order to reveal a possible correlation with the amount of daily diuresis: estimated GFR (eGFR calculated with MDRD formula), age, gender, primary renal disease, presence/ absence of systemic hypertension, presence/ absence of systolic cardiac dysfunction (defined as ejection fraction (EF) of left ventricle < 50% in echocardiography), presence/ absence of severe malnutrition (defined as serum albumin < 2.5 g/ dL), modality of initiating dialysis (emergency or programmed), type of vascular access (temporary or definitive).

Statistic interpretation of the results was performed by using T, Z tests, and Excel.

## Results

When starting HD, 76 patients had a urine output ≤ 500 mL/ day (group A), 84 had between 500–1200 mL/ day (group B), and 56 had ≥ 1200 mL/ day (group C). The characteristics of the patients are listed in **Table 1**.

**Table 1:** Characteristics of patients in the three groups

								Primary renal disease			
GROUP (N)	Median diuresis (mL/ day)	Median age (years)	Gender	> 65 years	Average eGFR (mL/min/1,73m2)	DN	GN	CTIN	VN	CD	U
A (76)	298.68	61.5 (21-78)	47 M 29 F	37	10.306	33	19	5	12	1	6
B (84)	872.62	619 (29-78)	55 M 29 F	43	10.251	16	4	12	43	7	2
C (56)	1759.82	61.7 (34-78)	30 M 26 F	30	10.273	9	2	20	12	13	0
*Legends: M = male; F = female; DN = diabetic nephropathy; GN = glomerular diseases (non-diabetic); CTIN = chronic tubulointerstitial nephropathies; VN = vascular nephropathies; CD = cystic diseases; U = unknown*											

**Fig. 1:**
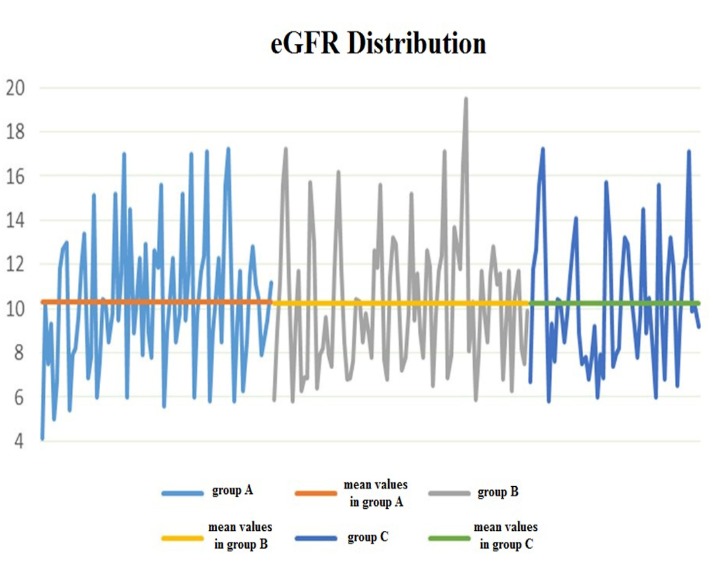
Mean values distribution of eGFR in the three groups of patients (T test)

No significant relation between eGFR and urine daily volume was found (T test – **Fig. 1**, **Table 2**.). For example, for an eGFR (MDRD formula) of 6 mL/min/1,73m2, we had patients with a daily diuresis of 0, 500, 1400 and 3200 mL/ day, respectively.

**Table 2:** Statistic interpretation in the three groups of patients

Groups of patients	Average	SD	*n*
Group A	*X A = 10.31*	*S A = 3.12*	*n A = 76*
Group B	*X B = 10.25*	*S B = 3.05*	*n B = 84*
Group C	*X C = 10.37*	*S C = 2.91*	*n C = 56*
Group A vs. Group B	*Sd = 9.52*	*T = 0.11*	*p = 0.9109*
Group B vs. Group C	*Sd = 8.98*	*T = 0.04*	*p = 0.9661*
Group A vs. Group C	*Sd = 9.22*	*T = 0.06*	*p = 0.9513*
	t n1+n2-2;1-α=1.30		

**Fig. 2:**
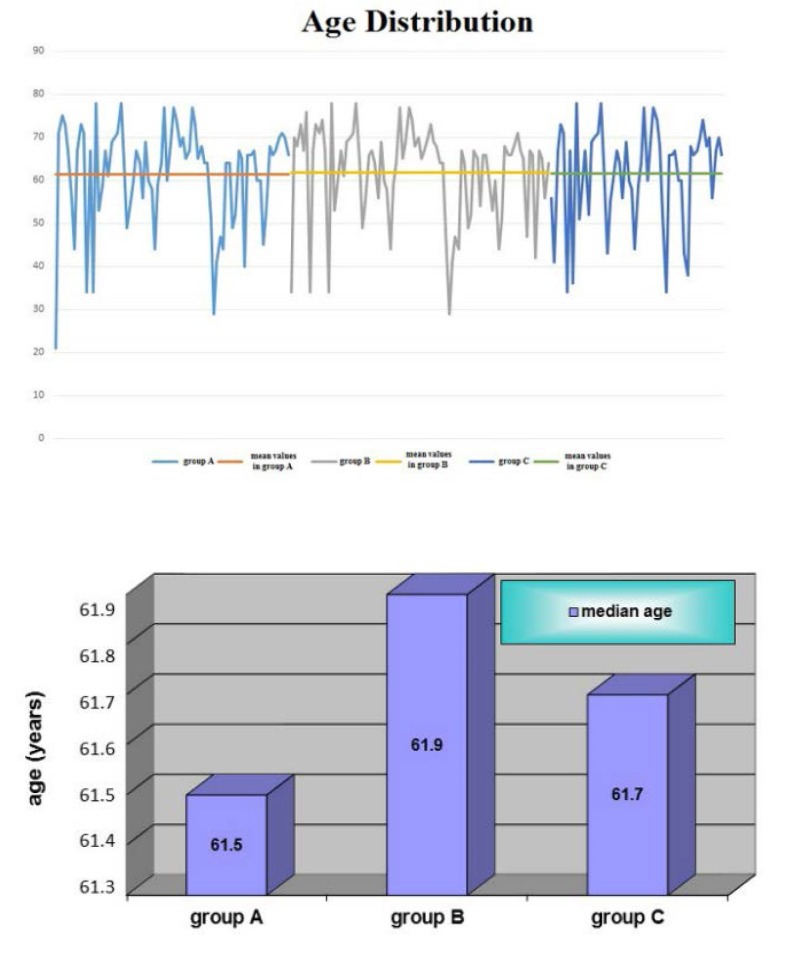
Age distribution in the three groups – no relationship between age and residual diuresis was found significant

There were no significant difference regarding the median age in the three groups (*T test, p between 0.8233 and 0.9212*) – **Fig. 2**, **Table 3**

**Table 3:** Statistic interpretation in the three groups of patients

Groups of patients	*Sd*	*T*	*p*
Group A vs. Group B	*130.7*	*-0.22*	*0.8233*
Group A vs. Group C	*124.0*	*0.10*	*0.9212*
Group B vs. Group C	*136.9*	*-0.10*	*0.9173*
	t n1+n2-2;1-α=1.30		

**Fig. 3:**
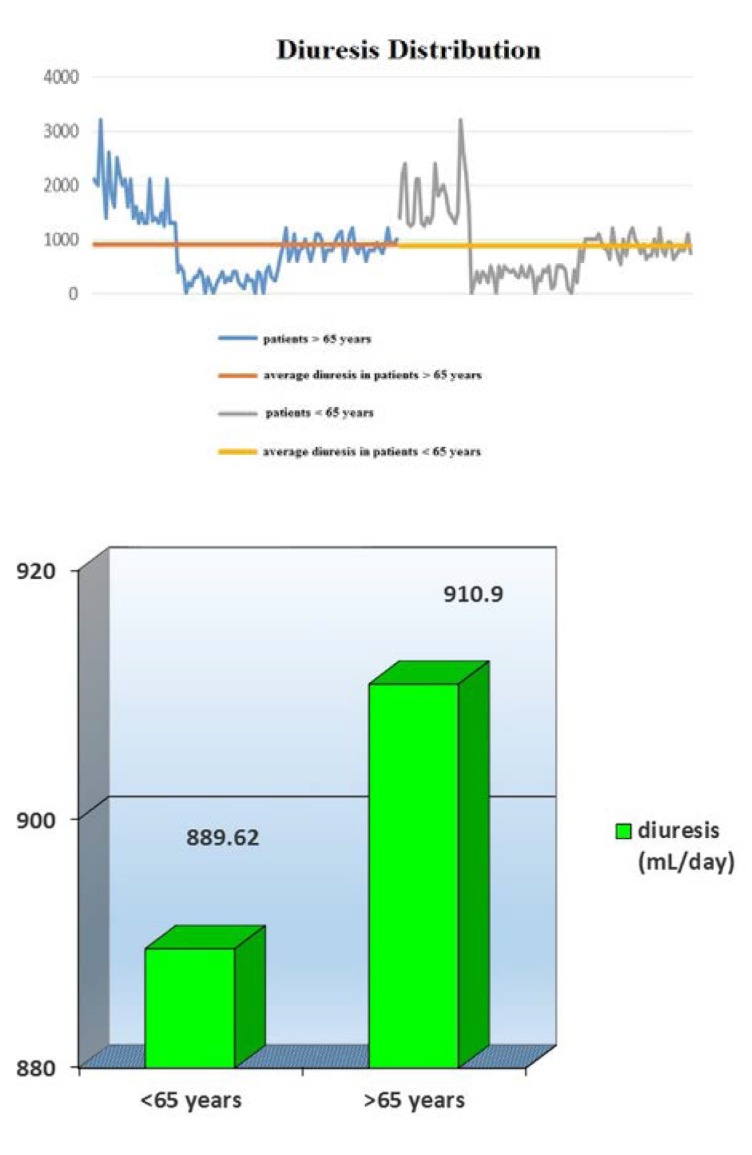
Average diuresis in patients < 65 years and > 65 years

In addition, statistical analysis revealed no significant differences between residual urine output in patients > 65 years (average 910.9 mL/ day) *versus* those < 65 years (average 889.62 mL/ day) (*T test, p= 0.8062*) – **Fig. 3**.

**Fig. 4:**
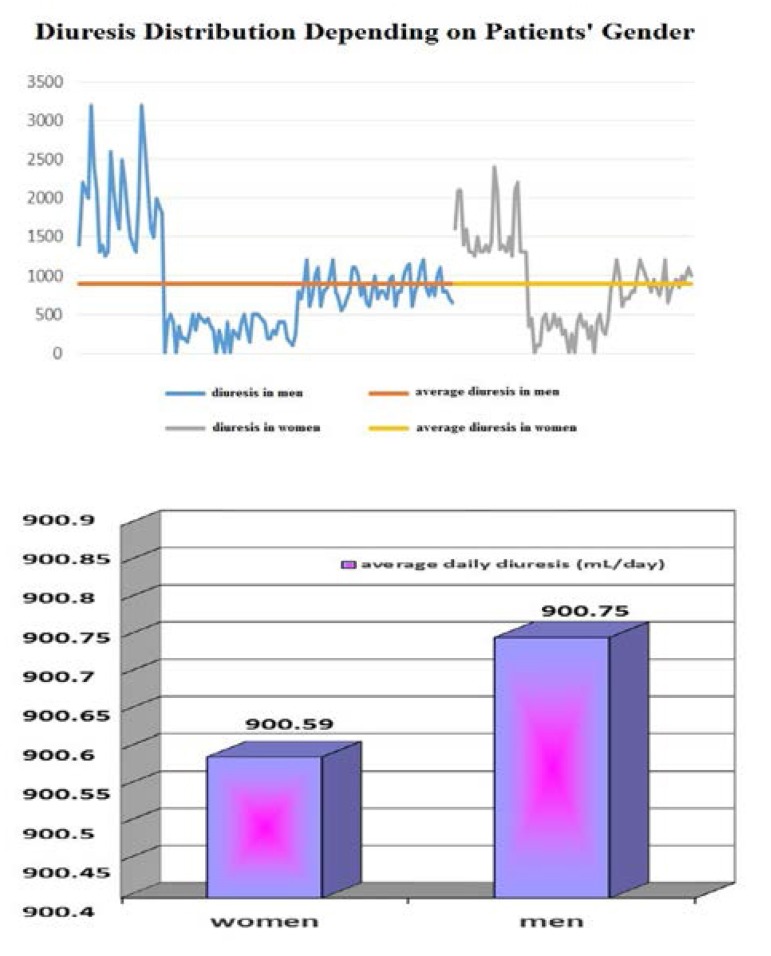
Average diuresis in men and women

Male gender prevailed in all three groups; no relationship between average diuresis and gender was found when statistical analysis was performed (*T test, p = 0.9985*) – **Fig. 4**.

**Fig. 5:**
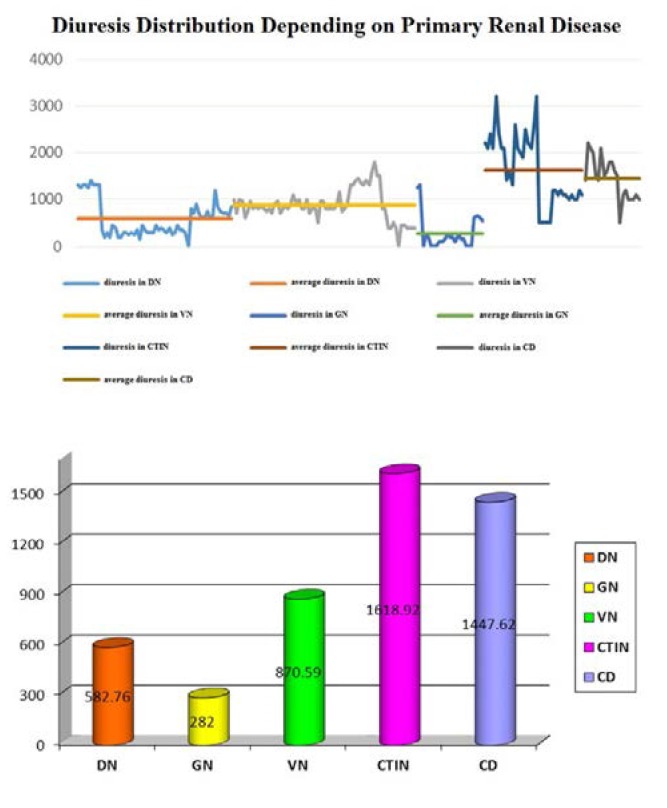
Average diuresis volume depending on primary renal diseases *Legends: DN = diabetic nephropathy; GN = glomerular diseases (non-diabetic); CTIN = chronic tubulointerstitial nephropathies; VN = vascular nephropathies; CD = cystic diseases*.

Analyzing the primary renal diseases in the three groups, we noted that chronic non-diabetic glomerulopathies, followed by diabetic nephropathies were associated with the lowest urine volumes: average of 282 mL/ day and of 582.76 mL/ day, respectively (*T test, p = 0.012*). Chronic tubulointerstitial nephropathies were correlated with increased urine output, average 1618.92 mL/ day (*T test, p = 0.3506*) – **Fig. 5**.

**Fig. 6:**
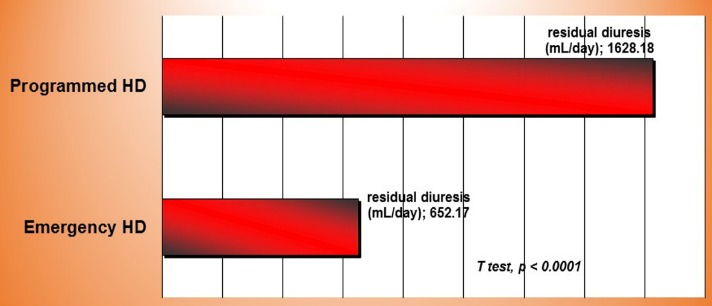
Residual diuresis in emergency and programmed initiation of hemodialysis

Residual diuresis was significantly higher in patients with programmed dialysis versus those who began the treatment in emergency (*T test, p < 0.0001*) – **Fig. 6**.

**Fig. 7:**
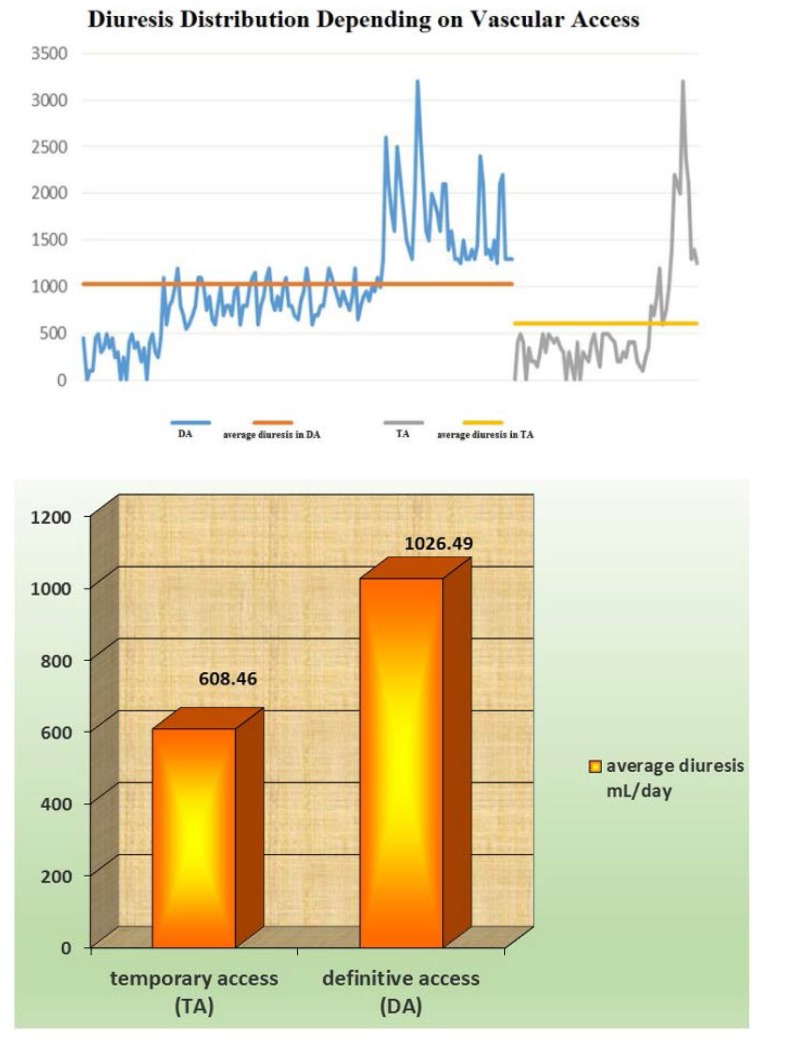
Average diuresis depending on the type of vascular access

Patients having already a mature vascular access at the time of HD initiation were proved to have a better urine volume preservation when compared with those beginning hemodialysis on temporary catheters (*T test, p = 0.00006*) – **Fig. 7**.

**Fig. 8:**
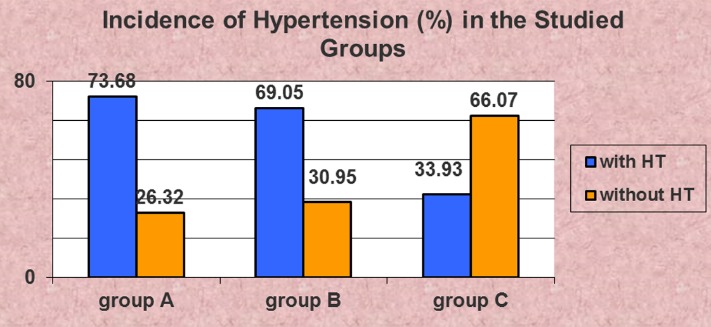
Incidence of systemic hypertension (HT) was not significantly different between groups A and B (*Z test*)

History of systemic hypertension did not significantly differ between group A and B (Z test, *Z = 0.69*), but as expected it was less frequent in group C (Z test, *Z = 6.01 group C versus group A, and Z = 6.01 group C versus group A, respectively*), as this group of patients included especially chronic tubulointerstitial nephropathies – **Fig. 8**.

**Fig. 9:**
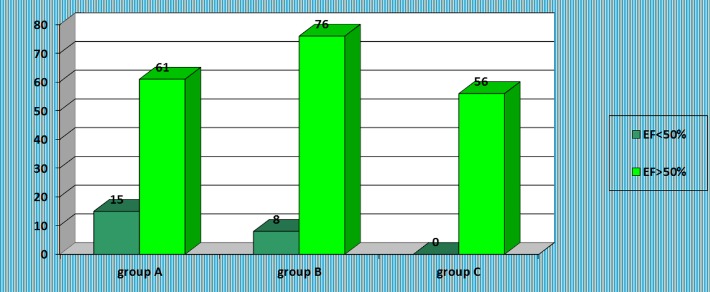
Presence of systolic dysfunction in the studied groups

Instead, statistical analysis revealed a strong relationship between systolic dysfunction and oliguria (*Z test, Z = 3.44*); 19.73% from all patients in group A, and 9.52% from patients in group B had reduced EF, while no patient in group C was recorded with systolic dysfunction – **Fig. 9**.

**Fig. 10:**
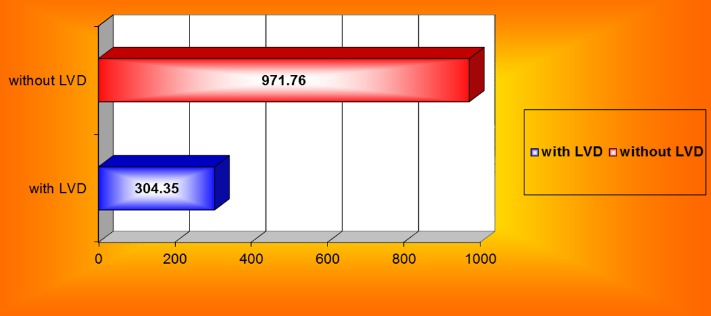
Average diuresis in patients with and without left ventricular systolic dysfunction (LVD)

Urine volumes were significantly lower in patients with systolic dysfunction, even in asymptomatic patients – **Fig. 10**.

**Fig. 11:**
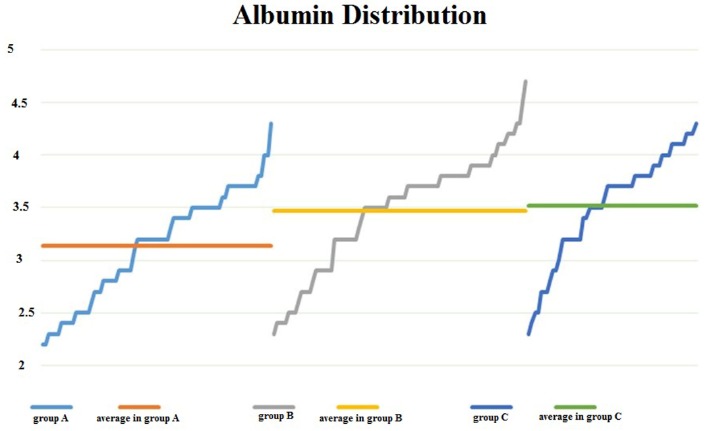
Albumin values were significantly lower in group A versus group B and C, respectively

**Fig. 12:**
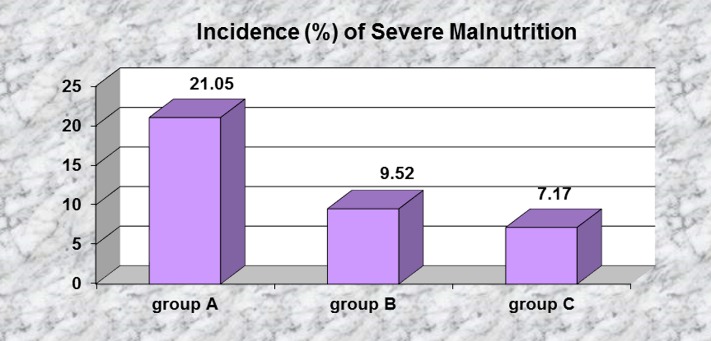
Severe malnutrition before initiating dialysis is significantly more frequent in group A

In patients from group A, plasma albumin values were lower than in the other two groups (*T test*, **Fig. 11**, **Table 4**), and severe malnutrition, defined by decreased plasma albumin < 2.5 g/ dL, was more frequent in group A when compared with the other patients (*Z test, Z = 1.50*) – **Fig. 12**.

**Table 4:** Statistic interpretation in the three groups of patients

Groups of patients	Average	SD	*p*
Group A	*X A = 3.14*	*S A = 0.52*	*n A = 76*
Group B	*X B = 3.47*	*S B = 0.55*	*n B = 84*
Group C	*X C = 3.52*	*S C = 0.52*	*n C = 56*
Group A vs. Group B	*Sd = 0.288*	*T = 3.94*	*p = 0.0001*
Group B vs. Group C	*Sd = 0.290*	*T = 0.48*	*p = 0.6316*
Group A vs. Group C	*Sd = 0.268*	*T = 4.16*	*p < 0.0001*
	t n1+n2-2;1-α=1.30		

## Discussions

In our research, we found no relationship between residual GFR and residual diuresis in patients with CKD stage 5 at the beginning of hemodialysis. This finding is in accordance with other studies which emphasize that, in uremic patients, diuresis is determined not only by residual GFR, but also by the rate of tubular reabsorption [**20**,**21**]. The equilibrium between GFR and tubular reabsorption is lost in advanced uremia, sometimes in earlier stages, depending on the concomitant complications as hypertension or/ and the primary renal diseases [**21**]. Preserving urine output in patients with chronic tubulointerstitial nephropathies supports the hypothesis that the tubular inability to reabsorb sodium and water is responsible for increased diuresis [**21**]. Later research revealed that the urine volume is less influenced by tubular inability to preserve sodium and water which may be reversed, at least partially, by low-sodium diet [**22**,**23**] and more by primary renal sodium retention with hypervolemia and by increased sanguine osmolarity as a result of urea and sodium retention [**21**,**24**]. It was proven that, in advanced chronic renal failure, there is an alteration of concentration and dilution tubular capacity as well as a result of tubulointerstitial fibrosis [**25**,**26**] and an increased tubular resistance at ADH [**21**,**24**,**27**,**28**]; as a consequence, in uremia, increasing daily water intake has a minor effect on diuresis.

No relation between age or gender and residual urine output was found in the present study. Regarding age, our results are not in accordance with literature data in which, in the majority of cases, age was a strong predictor of diuresis and/ or RRF loss [**29**], as a consequence of associated senile changes in the kidney [**29**-**32**]. This discrepancy might be explained by the fact that not all the studies used only diuresis as a measure for RRF. Regarding gender, literature offers conflicting data [**33**,**34**].

Although systemic hypertension has proved to be one of the most important progression factors of CKD [**25**], in our study, diuresis was not lower in hypertensive patients when compared with normotensive subjects. This observation might have at least two explanations:

- sodium retention and increased extracellular volume are the main cause of hypertension in advanced CKD [**25**,**26**]; increased retention of sodium is followed by increased natriuresis and polyuria, as an adaptive mechanism for reducing hypertension [**35**,**36**];

- use of diuretics is more frequent in patients with hypertension than in normotensives; increased diuresis may be, in these instances, drug-determined.

Additionally, the observation that hypertension per se does not alter diuresis confirms the fact that residual diuresis and residual GFR are not similar entities.

Presence of cardiac systolic dysfunction was significantly associated with lower urine output, as was severe hypoalbuminemia. Both these factors are associated with decreased renal flow secondary to reduced cardiac output in the first case and to lessened effective plasmatic volume in the second case. These observations are in accordance with literature data [**25**,**26**,**37**-**39**].

## Conclusions

Low GFR in CKD stage 5 patients is accompanied by different urine outputs. Systolic left ventricular dysfunction, glomerular etiology, malnutrition and late initiating of hemodialysis were proved to be associated with low residual diuresis in our study. Therefore, factors influencing residual diuresis may be distinct from those that influence residual GFR.

**Conflict of Interests**

The authors declare that there is no conflict of interests regarding the publication of this paper.
